# On Impact Damage and Repair of Composite Honeycomb Sandwich Structures

**DOI:** 10.3390/ma16237374

**Published:** 2023-11-27

**Authors:** Hang Zhang, Xiaopei Wang, Zhenhan Guo, Yuan Qian, Yan Shang, Deng’an Cai

**Affiliations:** 1 State Key Laboratory of Mechanics and Control for Aerospace Structures, Nanjing University of Aeronautics and Astronautics, Nanjing 210016, China; 2 Nanjing Research Institute on Simulation Technique, Nanjing 210016, China; 3 Purple Mountain Observatory, Chinese Academy of Sciences, Nanjing 210033, China

**Keywords:** honeycomb sandwich composites, impact energy, delamination damage, repair technique, strength recovery rate

## Abstract

This study is conducted on glass fiber-reinforced composite honeycomb sandwich structures by introducing delamination damage through low-velocity impact tests, establishing a three-dimensional progressive damage analysis model, and evaluating the delamination damage characteristics and laws of honeycomb sandwich structures under different impact energies through experiments. Repair techniques and process parameters for delamination damage are explored. It is found that as the impact energy increases, the damage area of honeycomb sandwich panels also increases, and the delamination damage extends from the impact center to the surrounding areas, accompanied by damage such as fiber fracture and matrix cracking. The strength recovery rates of sandwich panels at impact energies of 5 J, 15 J, and 25 J after repair are 71.90%, 65.89%, and 67.10%, respectively, which has a considerable repair effect. In addition, a progressive damage model for low-velocity impact on the composite honeycomb sandwich structure is established, and its accuracy and reliability are verified.

## 1. Introduction

Structural lightweight is the eternal theme in the development of aerospace structures. Composite honeycomb sandwich panels are widely used in the primary and secondary load-bearing structural elements of aircrafts by virtue of their superior properties such as high specific strength, high specific stiffness, and strong instability resistance [1]. However, it is easier to cause panel depression, delamination, core debonding, and even perforation damage when composite honeycomb sandwich structures are impacted by external objects in service [2,3,4,5,6]. In addition, the cost of resin matrix composites used in aircraft manufacturing is usually expensive. Hence, replacing or duplicating the damaged composite parts would result in a huge waste of funds and resources.

The emerging repair technology can cost-effectively solve the problem of significant decrease in flexural stiffness and structural strength of damaged composite honeycomb sandwich structures. The damaged composite parts can be well restored to their original performance and service life through an appropriate repair process. However, the composite honeycomb structure is characterized by complex structural form, large dispersion of mechanical properties, and various damage modes, which brings difficulty to strength-checking and repair program design. After the repair of the composite sandwich structure, different types of damage from the original ones may occur in the composite panel, adhesive layer, and honeycomb core layer. The mechanical properties such as the strength restoration effect of the repaired structure are important references to check whether the composite structure of the airplane can meet the airworthiness requirements. Therefore, combining with the engineering practice to reasonably assess the damage of composite sandwich structures before repair, proposing better repair methods, as well as predicting and evaluating the damage and strength of the repaired structure have become urgent issues in composite research, which are of great significance to further promote the application of composites in the field of aerospace [7].

For composite honeycomb sandwich structures, Avery et al. [8] and Hansen [9] investigated the lateral compression performance of sandwich panels containing panel core debonding damage using both experimental and numerical methods. The results showed that the core cannot be ignored in the case of buckling of a debond nor can it be simulated in terms of simple boundary conditions. Liu et al. [10] used three methods to repair composite honeycomb sandwich structures and investigated lateral compressive behavior of scarf-repaired honeycomb sandwich panels experimentally and numerically. The results showed that different repair methods affected the load-carrying capacity of the sandwich panel, and the repair design should be as symmetrical as possible to avoid additional bending moments. Campilho et al. [11] used a three-dimensional (3D) numerical model with a cohesive damage element to evaluate the tensile strength of CFRP-laminated composite repaired by external adhesives and investigated the effect of geometry on stress distribution and structural strength. The results showed that the repair strength increased exponentially with decreasing miter angles. For smaller miter angles, shear behavior had a greater effect on the strength than peeling behavior. Kashfuddoja et al. [12] analyzed the full-field strain changes in notched and repaired panels using the 3D-DIC technique and numerical simulations. It was found that the damage in the panels always started with matrix cracking around the hole and extended perpendicular to the hole edge. Wang et al. [13] found that the stacking sequence, plywood thickness, and adhesive strength had an effect on the strength of the repair due to the variation in stiffness along the repair zone. They also concluded that the design of stepped gouge repair was comparable to the performance of diagonal-jointed gouge repair due to the similarity of the inherent stress concentration at the edge of the repair zone in both repair methods. Wang et al. [14] investigated the repair methods of unilateral gouging, unilateral-lining gouging, and double-sided patching for damaged composite honeycomb sandwich structures and came up with the changing law of stiffness and strength of repaired composite honeycomb sandwich structures. Guo et al. [15] investigated the flexural properties of honeycomb sandwich composites after gouge repair using the three-point bending test. Test data proved the possibility of the gouge repair process. Then, a 3D finite element model was also established, and the damage initiation and evolution of honeycomb materials were realized by writing a user-defined field variable subroutine VUSDFLD. Their numerical simulation results were consistent with the test results. Yang et al. [16] used a combination of experimental testing and analytical simulation to investigate the effect of the dug-patch repair parameters on the bending strength of T300/QY8911 honeycomb sandwich structures and to determine the optimum dug-patch repair slope. The results showed that the repair slope had an important effect on the bending strength of the gouge repair specimens of the honeycomb sandwich structure and the smaller the repair slope, the larger the overlap area of the patch and the more pronounced the effect of the patch. The Kriging model was also used to optimize the design of the composite repair structure, and the resulting analytical model was accurate and reliable, with an error of less than 4.99%. Ramantani et al. [17] developed a cohesive zone model (CZM) for mixed type I + II damage at the adhesive interface layer, which was used to simulate and calculate the damage characteristics of patch repair and gouge repair structures under bending load. The main geometric parameters related to good repair performance, i.e., patch lap length and patch thickness for patch repair and patch slope for gouge repair, were investigated from the perspective of stress analysis and strength prediction. Zhao et al. [18] took the composite honeycomb structure and its typical glued patch repair structure as the research object and used the sandwich structure theory and the separated solid modeling method to establish a 3D progressive damage analysis finite element model to study the progressive damage assessment method and the influence factors of the repair effect of the patch repair numerically.

This paper focused on the panel damage identification and repair of glass fiber-reinforced composite honeycomb sandwich structures. The initial damage in the composite honeycomb sandwich structure is introduced by a drop hammer impactor to establish a three-dimensional progressive damage analysis model. The finite element numerical simulations and tests for low-velocity impact are performed to evaluate the delamination damage characteristics and laws of honeycomb sandwich structures under different impact energies. The sensitivity of the damage to the impact energy is studied. The repair technology and process parameters for the delamination damage to improve the strength of the composite honeycomb sandwich structures after repairing are also researched and discussed.

## 2. Materials and Manufacturing Process

The raw materials used to manufacture the specimens are shown in Table 1. The glass plain fabrics are provided by Nanjing Fiberglass Research & Design Institute Co., Ltd., Nanjing, China. The Nomex aramid paper honeycomb with a hole edge length of 4.8 mm is provided by EasyComposites Co., Ltd., Beijing, China. The mechanical parameters of the Nomex aramid honeycomb core are shown in Table 2. *E*_TT_ is the elastic modulus in the T-direction of the honeycomb core; *G*_LT_ and *G*_WT_ are the shear moduli in the LT and WT directions, respectively; *X*_TT_ is the tensile and compressive strength in the T direction; *S*_LT_ and *S*_WT_ are the shear strengths in the LT and WT directions, respectively; the subscript L represents the length direction of the honeycomb panel, W represents the width direction, and T represents the height direction.

**Figure 1:**
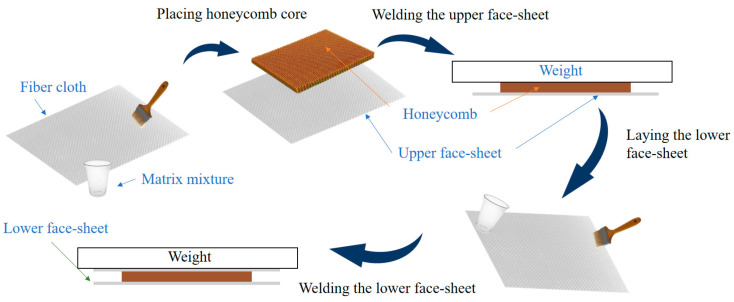
Manufacturing process.

The hand lay-up process was used to manufacture the test specimens. The manufacturing process of specimens is shown in Figure 1. We saturated the liquid resin matrix with fiberglass scrim fabric using a brush or roller and then laid the fabric with matrix down layer by layer according to a preset angle with a stacking sequence of [(0, 90)/(±45)]s. We placed the cut Nomex aramid honeycomb core on the uncured panel. Since the resin was fluid for a long time during the hand stack molding process, we applied pressure to eliminate gaps between the core layer and the panel. During the curing process, a weight of 5 kg was added to the panel.

After the panel is fully cured, the core layer will naturally bond to the panel. In addition, all panels were extruded with a rigid smooth board of the same weight before placement of the core layer. Rigid matting with a thickness of 1 mm was laid around the perimeter to ensure that the panels were made with the same fiber content each time and air bubbles were expelled. After there was no resin outflow, the rigid panels were removed. Finally, the other side of the panel was completed in the same manner. The advantage of this preparation method is that it ensures a strong bond between the core layer and the face sheets, and the uncured panel is always in the lower part during the molding process to avoid the resin flowing into the pores of the core layer. After the molding was completed, the specimens were cut according to the standard to 150 mm × 100 mm.

## 3. Impact Test and Simulations

### 3.1. Low-Velocity Impact Test

#### 3.1.1. Experimental Procedures

**Figure 2:**
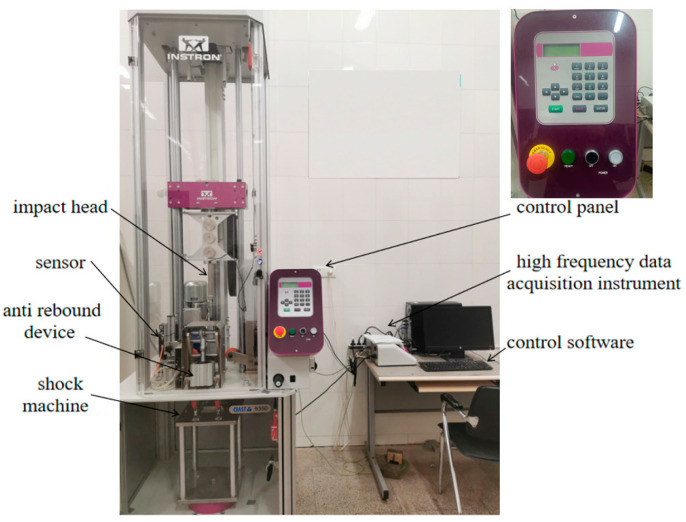
Impact test machine.

The low-velocity impact tests on the honeycomb sandwich structures were carried out using the INSTRON CEAST 9350, CEAST Headquarters, Vicenza, Italy, which consists of a test bench, spare parts box, electrical cabinet, and control instrumentation, as shown in Figure 2. The front end of the impactor is a semi-circle with a diameter of 16 mm and a mass of 5.277 kg.

**Figure 3:**
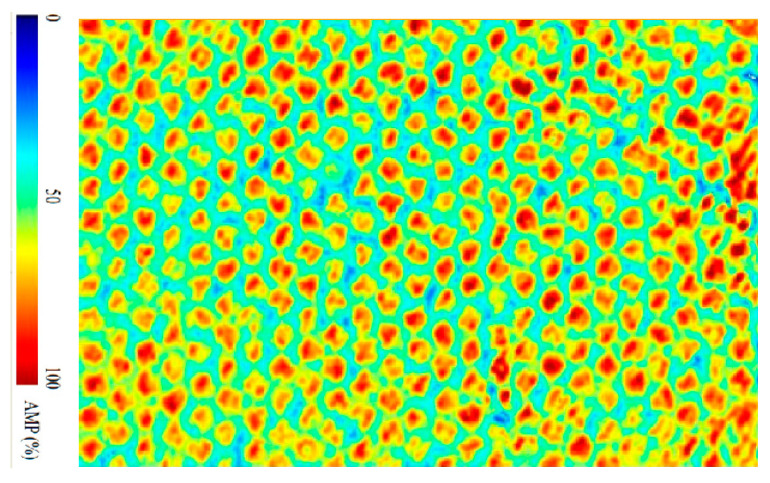
Ultrasonic scanning result of no initial damage in specimen.

Prior to the low-velocity impact test, all specimens were subjected to an ultrasonic C-scan to ensure that no initial damage existed in specimens. The C-scan results indicated that no significant initial damage existed in any of the specimens and that the test requirements were met. The ultrasonic scanning result is shown in Figure 3. The impact tests were conducted in accordance with ASTM D7136 [19]. During the impact test, we ensured that the impact location was the center of the upper surface of the specimen. Four restraints on the test platform were tightly compacted to ensure that the specimen did not move laterally under the impact force during the test. Pre-tests were carried out before the formal tests to determine the impact energy. The impact energy range was between 5 J and 30 J, such that penetration damage did not occur in the specimen after being impacted by the impactor, since the main objective of this paper is to study the repair techniques for delamination-damaged sandwich structures. Test results showed that the upper panel was damaged and the core layer was undamaged under impact energies of 2 J–20 J, and the upper panel was penetrated and the core layer was damaged under impact energies of 20 J–30 J. Therefore, impact energies of 5 J, 15 J, and 25 J were used for the formal low-velocity impact tests. For the three groups of tests, all specimens were cut from the same batch of panels to facilitate the comparison of the impact behavior under three different impact energies.

#### 3.1.2. Test Results and Discussion

**Figure 4:**
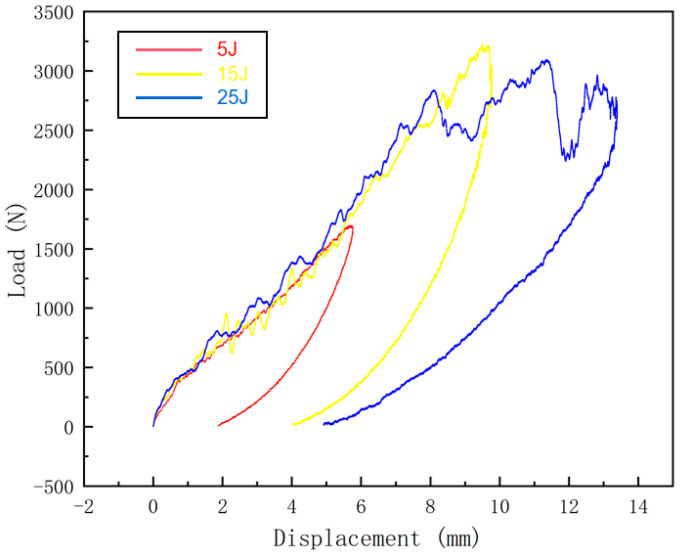
Load–displacement curves at different impact energies.

The raw data exported from the testing machine were processed to obtain the load–displacement curves at different impact energies, as shown in Figure 4. Analysis of the load–displacement curves shows that the load oscillates at the time of impact, which may be caused by the occurrence of fiber breakage, matrix cracking, and other damages to the sandwich panel. The load–displacement curve at 25 J of impact energy has two more obvious load drops during the descent of the punch as compared with the ones at 5 J and 15 J of impact energies. The first dropout occurs after the punch breaks through the upper panel, and then the load picks up. The second dropout occurs after the punch crushes the honeycomb core layer. The load fallback phase corresponds to the punch rebound. The area of the load–displacement curve is the energy absorbed by the sandwich panel.

**Figure 5:**
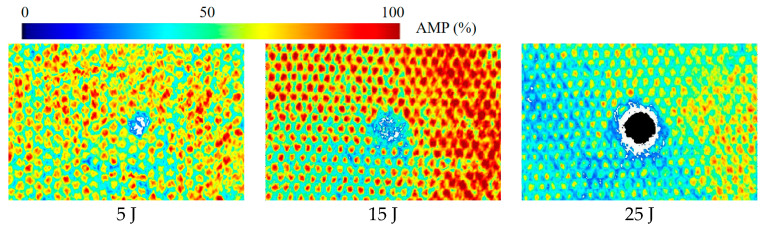
C-scan results of damaged specimens.

Damage to the sandwich panels after low-velocity impact tests is difficult to recognize by the naked eye and thus often observed by other means. Ultrasonic C-scans were performed on the specimens after the impact test to determine the area of delamination damage and the impact damage pattern of the honeycomb sandwich structure. The C-scan results are shown in Figure 5. It can be seen that both the damage form of sandwich panels impacted by different energies and the damage area are different. With the increase in impact energy, the delamination area gradually increases. Further, the panel shows different forms of damage, such as fiber fracture and matrix cracking. The upper facesheet of the sandwich panel impacted by 5 J of energy is slightly damaged, while a small amount of fiber fracture and matrix cracking are seen. Under 15 J of impact energy, the upper facesheet shows extensive matrix damage and some broken fibers. Besides, small delamination and honeycomb core damages are also observed. Under 25 J of impact energy, the upper facesheet of the sandwich specimen is penetrated, while a large number of fibers are broken, the matrix is cracked in large areas, the delamination damage is more serious, and the honeycomb core is crushed.

### 3.2. Finite Element Simulation

#### 3.2.1. Simulation Model

**Figure 6:**
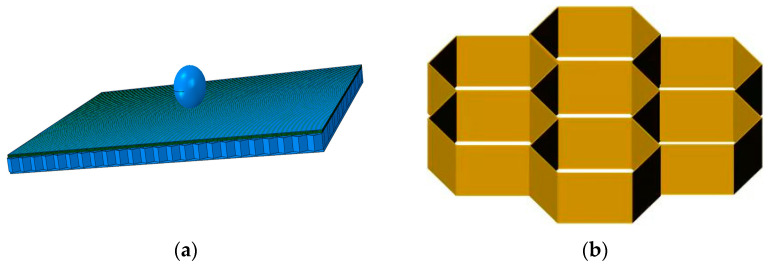
Finite element model. (**a**) simulation model. (**b**) cellular model.

The low-velocity impact process was numerically simulated using ABAQUS v6.14 [20]. A model was established according to the real size of the specimen, and the punch in the low-velocity impact test was modeled by a small ball endowed with an initial velocity. The simulation model is shown in Figure 6a. For modeling the honeycomb core, the desired honeycomb dimensions were quickly obtained in the form of a hexagonal array. The honeycomb core model is shown in Figure 6b. For comparison with the experiment, the same impact position as the experimental one was chosen. The variation in impact energy was controlled by the impact velocity.

**Figure 7:**
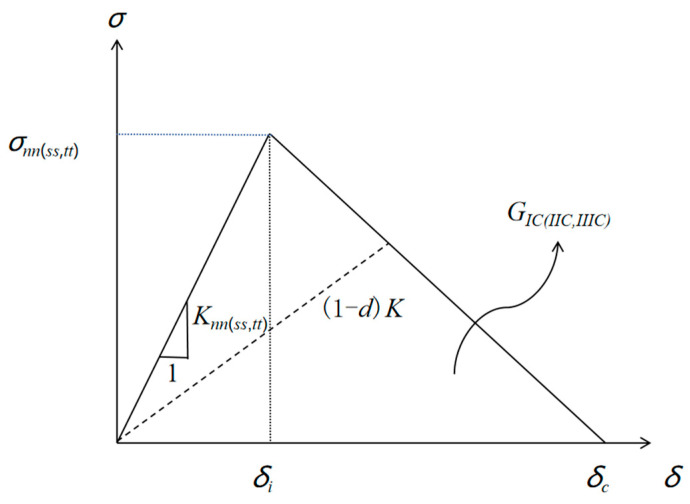
Cohesive constitutive relationship.

For the adhesive layer between the interfaces, cohesive units were inserted between the layers to simulate the interfacial delamination damage. The cohesive intrinsic relationship is shown in Figure 7. The interface mechanical properties are listed in Table 3.

#### 3.2.2. Failure Criteria

The fiber bundles constituting the core material can be regarded as unidirectional panels, and in this paper, the panels are regarded as composites with only one layer of fabric layup. Cheng et al. [21] and Yang [22] developed a failure criterion applicable to fabric materials on the basis of the 3D Hashin criterion by considering that both the warp and weft directions of the fabric material are mainly fiber bundles. The specific forms are as follows.

Weft fiber stretch failure ($\sigma_{11}>0$):

(1) $$F_{weft,ft}={\left(\frac{\sigma_{11}}{X_{t}}\right)}^{2}+\frac{1}{S_{12}^{2}}\left(\tau_{12}^{2}+\tau_{13}^{2}\right)=1$$

Weft fiber compression failure ($\sigma_{11}<0$):

(2) $$F_{weft,fc}={\left(\frac{\sigma_{11}}{X_{c}}\right)}^{2}=1$$

Warp fiber tensile failure ($\sigma_{22}>0$):

(3) $$F_{warp,ft}={\left(\frac{\sigma_{22}}{Y_{t}}\right)}^{2}+\frac{1}{S_{12}^{2}}\left(\tau_{12}^{2}+\tau_{13}^{2}\right)=1$$

Warp fiber compression failure ($\sigma_{22}<0$):

(4) $$F_{warp,fc}={\left(\frac{\sigma_{22}}{Y_{c}}\right)}^{2}=1$$

where $\sigma_{ij}(i,j=1,2,3)$ is the component of the stress tensor, $X_{t}$ and $X_{c}$ are the tensile and compressive strengths in the weft direction, $Y_{t}$ and $Y_{c}$ are the tensile and compressive strengths in the warp direction, $S_{12}$ is the in-plane shear strength, $S_{13}$ and $S_{23}$ are the out-of-plane shear strengths, respectively. Fiber failure occurs when $F_{weft,ft}$, $F_{weft,fc}$, $F_{warp,ft}$, or $F_{warp,fc}$ reaches one.

#### 3.2.3. Damage Evolution

When damage occurs to the material, the material properties begin to degrade. In this paper, the bilinear ontological relationship based on equivalent strain is adopted. The expression is shown below:

(5) $$d_{I}=\frac{\varepsilon_{eq,I}^{f}(\varepsilon_{eq,I}-\varepsilon_{eq,I}^{0})}{\varepsilon_{eq,I}(\varepsilon_{eq,I}^{f}-\varepsilon_{eq,I}^{0})},I\in \left(ft,fc\right)$$

(6) $$\varepsilon_{eq,I}^{}=\sqrt{\varepsilon_{1,I}^{2}},I\in (ft,fc)$$

where $\varepsilon_{eq,I}$ is an isotropic transformation, $\varepsilon_{eq,I}^{0}$ is the equivalent initial damage strain, and $\varepsilon_{eq,I}^{f}$ is the equivalent destructive strain. The equivalent initial damage strain is determined by the damage criterion and corresponds to the stress–strain values in Equations (1)–(4). Therefore, it can be obtained by equivalent strain,

(7) $$\varepsilon_{eq,I}^{0}=\varepsilon_{eq,I}^{}/F_{I},I\in (ft,fc)$$

For fiber damage, the equivalent failure strain can be calculated from the fiber fracture energy, i.e.,

(8) $$\varepsilon_{eq,I}^{f}=\frac{2G_{ft(c)}}{X^{t(c)}L}$$

where *L* is the characteristic length of the mesh in the finite element model, $G_{ft}$ is the fiber tensile breaking energy, and $G_{fc}$ is the fiber compression fracture energy.

The stiffness matrix of the core fiber bundle and panel fabrics after damage is updated using Equations (9) and (10):

(9) $$dC=\left[\begin{array}{cccccc} dC_{11} & dC_{12} & dC_{13} & 0 & 0 & 0\\ dC_{12} & dC_{22} & dC_{23} & 0 & 0 & 0\\ dC_{13} & dC_{23} & dC_{33} & 0 & 0 & 0\\ 0 & 0 & 0 & dG_{12} & 0 & 0\\ 0 & 0 & 0 & 0 & dG_{23} & 0\\ 0 & 0 & 0 & 0 & 0 & dG_{13} \end{array}\right]$$

(10) $$\begin{array}{l} d_{f}=1-(1-d_{ft})*(1-d_{fc})\\ dC_{11}=(1-d_{f})*C_{11}\\ dC_{22}=(1-d_{f})*(1-d_{mt})*(1-d_{mc})*C_{22}\\ dC_{33}=(1-d_{f})*(1-d_{mt})*(1-d_{mc})*C_{33}\\ dC_{12}=(1-d_{f})*(1-d_{mt})*(1-d_{mc})*C_{12}\\ dC_{13}=(1-d_{f})*(1-d_{mt})*(1-d_{mc})*C_{13}\\ dC_{23}=(1-d_{f})*(1-d_{mt})*(1-d_{mc})*C_{23}\\ dG_{12}=(1-d_{f})*(1-S_{mt}*d_{mt})*(1-S_{mc}*d_{mc})*G_{12}\\ dG_{13}=(1-d_{f})*(1-S_{mt}*d_{mt})*(1-S_{mc}*d_{mc})*G_{13}\\ dG_{23}=(1-d_{f})*(1-S_{mt}*d_{mt})*(1-S_{mc}*d_{mc})*G_{23} \end{array}$$

where *d* is the damage factor, the subscript *f* represents the longitudinal direction of the fiber bundle and the weft direction of the panel fabric, m represents the transverse direction of the fiber bundle and the warp direction of the panel fabric, and the default value of the damage factor is zero. Considering different effects of tensile and compressive damage on the shear properties of the material, the shear influence factors *S_mt_* and *S_mc_* are introduced accordingly, and the values of *S_mt_* and *S_mc_* are 0.90 and 0.50, respectively.

### 3.3. Numerical Results and Validation

#### 3.3.1. Model Validation

**Figure 8:**
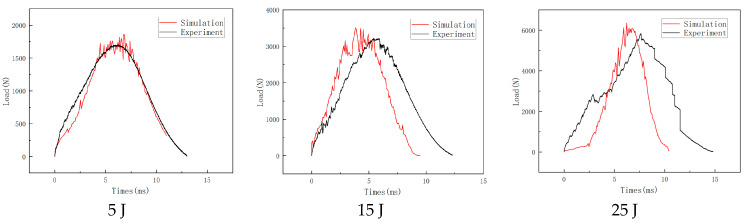
Load–time curves at different impact energies.

The numerical simulation results of ABAQUS are compared with the test data to validate the model, as shown in Figure 8. The comparisons show that the test and the simulation data are in good agreement, and the peak loads corresponding to different impact energies are basically the same. It is found that the load–time curve shows an oscillating upward trend in the initial stage of the impact. The slight load drop in the impacting process is due to the occurrence of damages in the panel area in contact with the punch, such as fiber breakage and matrix cracking. When the load is about to peak, the curve shows a high-frequency oscillation and the rate of load increase gradually slows down. Subsequently, the load gradually decreases, corresponding to the punch back up stage, consistent with the phenomenon in the impact test process. The difference between the simulation and experimental results is because the simulation did not simulate the generation of bubbles during the preparation process of the specimen. In addition, the honeycomb is composed of multiple closed hexagonal prisms. The air absorption and damping of the enclosed space are not considered in the simulation process, and the instability of the honeycomb is relatively complex, which resulted in deviations between the simulation and experimental results. Considering the uncertainties of the test data, the developed finite element model is reasonable and effective.

#### 3.3.2. Damage Analysis

**Figure 9:**
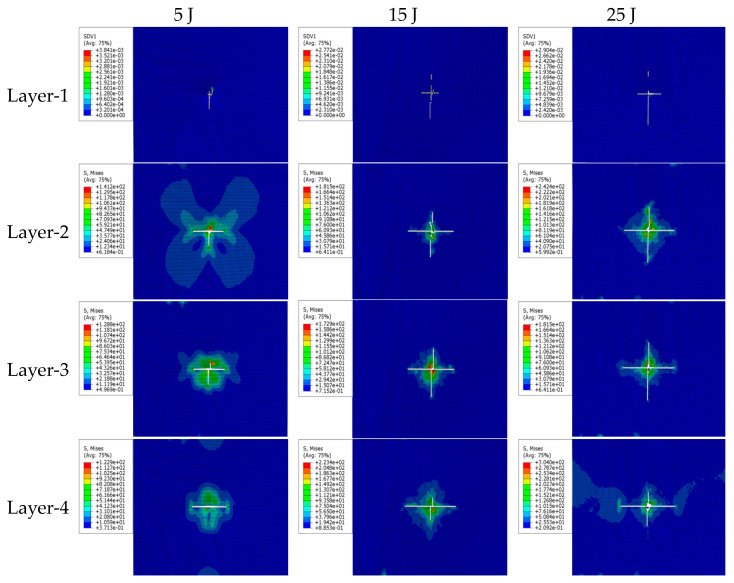
Damage nephogram at various impact energies.

**Figure 10:**
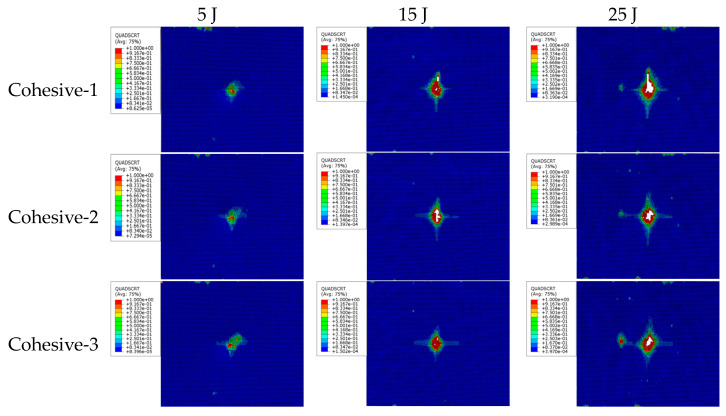
Cohesive damage nephogram.

Sandwich structures under low-velocity impact are often accompanied by typical damage patterns such as fiber breakage, matrix cracking, and delamination. However, it is difficult to observe and identify the damages during the impact test, and thus, numerical simulations are necessary, since identifying the damage patterns is very important for the safe design of the sandwich structures. The simulated damage at different impact energies is shown in Figure 9 and the simulated cohesive layer damage is shown in Figure 10, respectively.

In the damage cloud maps at different impact energies shown in Figure 9, Layer-1 to Layer-4 are the monolayers of the upper facesheet starting from the one closest to the impactor to one far away from the impactor, respectively. A large number of cells in each layer have matrix tensile damage, especially serious tensile damage in the single layer closer to the lower panel. Because of the bending deformation of the sandwich panel under impact loading, the stretching of the single layer away from the impactor is more serious, which makes the bottom layer have tensile failure first, and the damage gradually expands to the surface directly in contact with the impactor (impacted surface). The area of single-layer damage near the impacted surface is small, and the main damage modes are matrix extrusion and fiber extrusion damages. Comparisons of the same monolayer at different impact energies reveal that the damage to the monolayer is more severe and the damage area gradually increases with an increase in impact energy, consistent with the results of the impact test.

Analyzing the damage of the cohesive layer shown in Figure 10 reveals that the delamination damage occurs in each cohesive layer, and the delamination damage extends from the center of each cohesive layer to the surroundings with an irregular shape distribution. This is mainly due to the fact that the layup of the composite material is complex, and the stiffness of each layer in the same direction is different. Comparing the same cohesive layer under the same impact energy shows that the delamination area of the layer close to the punch is slightly smaller than the one of the layer far from the punch. Comparing the same cohesive layer under different impact energies shows that the delamination damage area increases with an increase in impact energy. In the monolayer away from the impacted surface, matrix cracking along the fiber direction occurs first, followed by delamination damage. The area of matrix cracking is larger than the area of delamination damage, and the shape of delamination damage is similar to that of matrix cracking damage. One may conclude that matrix cracking is an important reason for inducing delamination damage in the layup near the backside of the impact. In other words, the further away from the impacted zone, the more serious the matrix cracking damage in a single layer and the larger the area of interlayer delamination.

## 4. Specimen Repair

### 4.1. Patch Program Design

**Figure 11:**
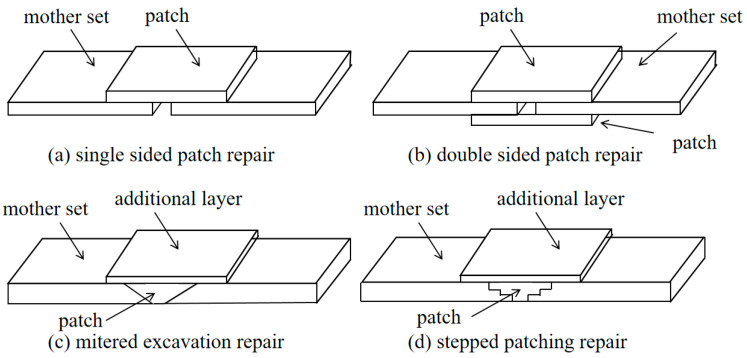
Typical adhesive repair types.

Repair studies of composite honeycomb sandwich structures focus on panel repair and honeycomb core layer repair. Core layer repair generally removes the damaged part of the core layer and replaces it with new honeycomb. Panel repair is mainly categorized into two types: patch-and-glue repair and gouge-and-glue repair. Patch repairs are generally used for thin structure repairs or temporary repairs, while gouge repairs are usually used for thicker structure repairs and permanent repairs. Gouging and gluing repairs are further divided into two types: diagonal gluing-gouging repairs and step gluing-gouging repairs [23,24]. With reference to the above-mentioned mature repair process of gluing gouge repair, the repair technology for delamination damage is investigated for molding repair of honeycomb sandwich structures after the introduction of impact damage. Typical types of gluing repairs are shown in Figure 11.

**Figure 12:**
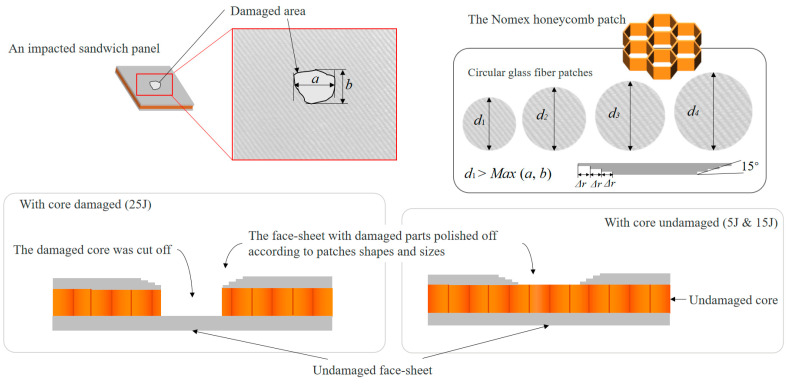
Repair schematic diagram.

Combined with the molding characteristics of the specimen and the form of damage, the stepped gluing gouge repair process is selected. The repair angle is determined to be 5°. The repair schematic is shown in Figure 12.

### 4.2. Evaluation of the Effectiveness of the Repair

**Figure 13:**
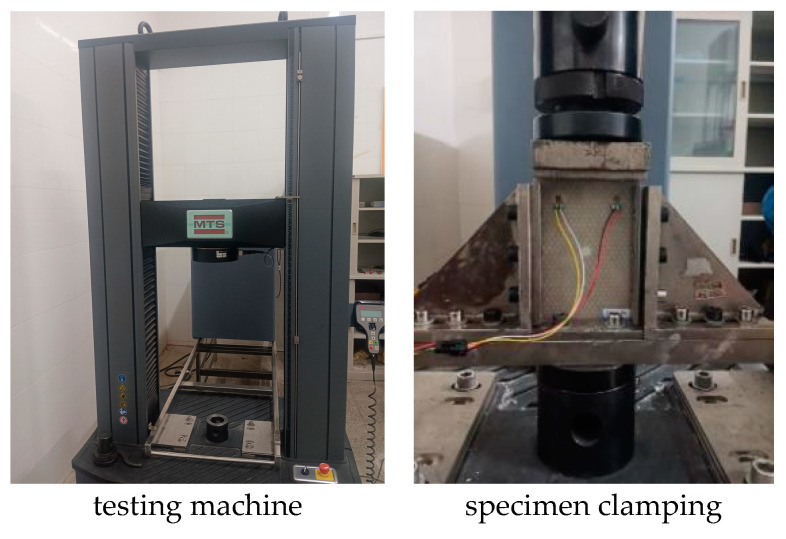
Compression test.

Compression tests were carried out on the intact parts, parts with damage introduced, and repaired specimens to measure their compressive strength. The compression test was carried out using an MTS static testing machine and the test standard was executed according to ASTM D7137M [25], and the compression test is shown in Figure 13.

The damage load of the repaired specimen was analyzed in comparison with the damage load of the intact and damaged pieces. The damage load of the intact part was 28.50 kN, and the strength recovery rate is shown in Table 4. The strength recovery rate is the ratio of the damage load of the repaired specimen to the damage load of the intact one. It can be seen that the strength of the repaired specimen is improved compared with that before repair, which verifies the feasibility of the repair method. And the final strength recovery rate can reach about 65% of the intact part, and the repair effect is more considerable. In addition, the strength recovery rate of the repaired specimen decreases with an increase in impact energy, and the size of the initial loss also has a certain effect on the repair effect. In addition, the honeycomb inside the sandwich structure was damaged differently due to different impact energies. At 5 J and 15 J of impact energy, the honeycomb was not damaged or only slightly damaged, and the honeycomb was not repaired during the repair process. Under 25 J of impact energy, the honeycomb is crushed and the damaged part of the honeycomb is removed and replaced with a new honeycomb during repair. Whether or not the honeycomb is repaired may also have an effect on the final strength recovery rate.

## 5. Conclusions

The composite honeycomb sandwich structures were fabricated using a hand lay-up molding process. A progressive damage model that can be applied to the low-velocity impact of the composite sandwich panels was established. The consistency between the simulation results and test data was verified, which confirms the accuracy and reliability of the model. The relevant mechanical responses and damage modes under different impact energies were also obtained. It was shown that with an increase in impact energy, the damage area of the specimen increases, accompanied with fiber fracture, matrix cracking, delamination, and other damage modes.

Compression tests were conducted on the intact, impact-damaged, and repair specimens. It was found that the compression strength of the repaired specimens was improved to a certain extent compared with that before repair. The strength recovery rates of sandwich panels at impact energies of 5 J, 15 J, and 25 J after repair were 71.90%, 65.89%, and 67.10%, respectively, which had a certain repair effect.

The low-velocity impact model established in this paper can effectively simulate the impact response and delamination damage of honeycomb sandwich panels. The failure modes such as honeycomb collapse and instability were not considered. A more accurate prediction model can be improved in the future. In addition, the repaired sandwich panel can also be subjected to multiple impacts to study the residual strength under more complex conditions.

## Figures and Tables

**Table 1:** List of raw materials.

Fiber Cloth	Matrix	Honeycomb Core
Glass plain fabric	E51 epoxy resin, benzene dimethylamine	Nomex aramid paper honeycomb

**Table 2:** Mechanical parameters of Nomex aramid honeycomb core.

*E*_TT_/MPa	*G*_LT_/MPa	*G*_WT_/MPa	*X*_TT_/MPa	*S*_LT_/MPa	*S*_WT_/MPa
140	40	25	2.4	1.2	0.7

**Table 3:** Interface mechanical properties.

Parameter Name	Numerical Value
*K_n_*_,_*G_s_*_,_*G_t_* (MPa)	3500
*t_n_*^0^, *t_t_*^0^ (MPa)	35
*t_s_*^0^ (MPa)	65
*G_n_^c^* (N·mm^−1^)	0.252
*G_s_^c^*, *G_t_^c^* (N·mm^−1^)	0.501
*η*	2

**Table 4:** Strength recovery rate.

Impact Energy/J	Damage Load before Repair/kN	Damage Load after Repair/kN	Strength Recovery Rate/%
5	16.10	20.50	71.90
15	12.56	18.78	65.89
25	12.01	17.90	67.10

## Data Availability

Data are unavailable due to privacy.
